# Diagnostic Accuracy of an Indirect Enzyme Linked Immunosorbent Assay (iELISA) for Screening of *Babesia bovis* in Cattle from West Africa

**DOI:** 10.3390/life13010203

**Published:** 2023-01-10

**Authors:** Alassane Toure, Moussa Sanogo, Abdelmalek Sghiri, Hamid Sahibi

**Affiliations:** 1 UFR Sciences de la Nature, Nangui Abrogoua University, P.O. Box 801 Abidjan 02, Côte d’Ivoire; 2 Laboratoire Central Vétérinaire de Bingerville, LANADA, P.O. Box 206 Bingerville, Côte d’Ivoire; 3 Institut Agronomique et Vétérinaire Hassan II, Madinat Al Irfane, P.O. Box 6202, Rabat 10101, Morocco

**Keywords:** *Babesia bovis*, cattle, iELISA, index test, Bayesian, RT-PCR

## Abstract

The epidemiology of corresponding tick-borne diseases has changed as a result of the recent introduction of *Rhipicephalus (Boophilus) microplus* to West Africa. The current study aimed to assess the diagnostic performance of an indirect ELISA for the detection of *Babesia bovis* infection in cattle. In a cross-section study, using a Bayesian Latent Class Model and iELISA diagnostic test for cattle babesiosis due to *Babesia bovis*, accuracy has been assessed with RT-PCR as an imperfect reference test. A total of 766 cattle were tested. The optimal diagnostic performances were obtained with 5% percentage of positivity. Sensitivity and specificity were, respectively, 0.94 [Cr. I.: 0.85–0.99] and 0.89 [Cr. I.: 0.87–0.92]. Additional diagnostic characteristics revealed that the Positive Predictive Value (PPV) and Negative Predictive Value (NPV) were 96.6% [Cr. I.: 92.7–100%] and 82.2% [Cr. I.: 72–93%]. Overall, this test well discriminates an infected status from an uninfected status considering the area under the ROC curve (AUC) which was 0.78 [Cr. I: 0.72–0.85] and a Diagnostic Odds Ratio (DOR) of 127.8 [Cr. I.: 10.43–1562.27]. The AUC was significantly higher than 0.5 (*p* < 10^−5^). In consequence, this serologic assay could be suitable in moderate to high prevalence assessments.

## 1. Introduction

Babesiosis is one of the most important tick-borne infections in cattle. It is caused by *Babesia bigemina* and *Babesia bovis*, the two species reported in West Africa [1]. While data are lacking on the actual economic impact of tick-borne diseases such as babesiosis in Africa and specifically in West Africa, they were reported to have caused losses of USD 3.10 million and USD 0.60 million in Indonesia and the Philippines [2], respectively, and USD 100 million and USD 6.7 millions per year in Australia [3]. One of the challenges in implementing a control programme is the ability to accurately identify infected animals from non-infected ones. During many years, diagnostic tests were developed for this purpose including Smear staining, Enzyme-Linked Immuno-Sorbent Assay (ELISA), Immuno-Fluorescent Antibody Test (IFAT), Polymerase Chain Reaction (PCR), or Real Time-PCR (RT-PCR). Since they are of key importance to estimating epidemiological indicators such as prevalence and incidence, knowledge on test characteristics such as sensitivity and specificity of diagnostic assays is crucial. 

Taking into account the recent introduction of the *Rhipicephalus* (*Boophilus*) *microplus* tick in West Africa [4,5], which is the main vector of *Babesia bovis* and more pathogen than *Babesia bigemina*, a great change in babesiosis (caused by *Babesia bovis*) prevalence (amongst other epidemiological parameters) is very possible. In order to control this disease, we need to assess disease importance or frequency accurately. Before this assessment, it is essential to evaluate the diagnostic test. Indeed, it is well-known that the ELISA test is better at testing a lot of samples and is cheaper than the molecular one. Competitive ELISA has been validated in many laboratories, but indirect ELISA has not. In fact, using whole merozoites antigen, iELISA has generally been proven to be weak because of varying threshold values between laboratories [6]. Therefore, it is relevant to evaluate with more key parameters beyond sensitivity and specificity in the field. Moreover, adding a Bayesian methodology in our work avoids overestimates of diagnostic test parameters. Therefore, the aim of the current study is to assess the performance of an indirect ELISA for detecting antibodies against *Babesia bovis* infection in cattle. 

## 2. Materials and Methods

### 2.1. Study Area and Data Collection 

**Figure 1:**
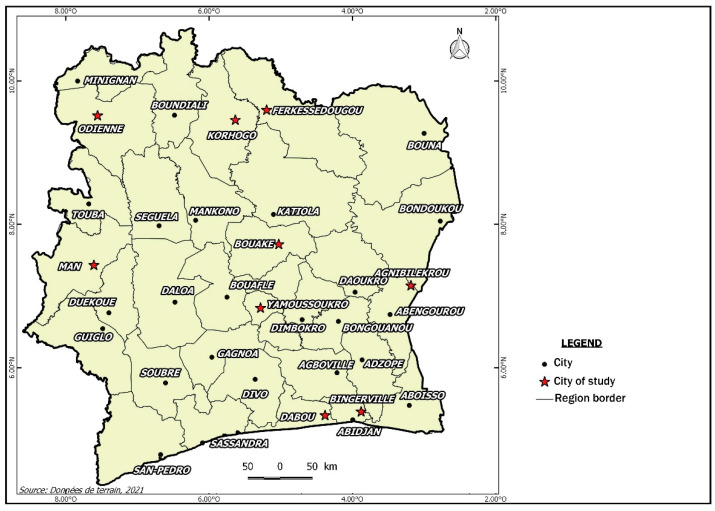
Study sites in Côte d’Ivoire.

The data used for this study were randomly collected in Côte d’Ivoire, a West African country, between February 2014 and March 2014 (Figure 1). In this country, cattle originating from areas infested by newly introduced *Rhipicephalus (Boophilus) microplus* were blooded. The cities involved in the study were Ferkessedougou, Korhogo, and Odienné in the north, Agnibilekrou city in the east, Man in the west, Yamoussoukro city in the centre, and Dabou and Abidjan in the south.

In addition, to get negative control sera, blood samples were also collected from 288 cattle rearing in Burkina Faso north areas that were free from *Rhipicephalus* (*Boophilus*) *microplus* and *Rhipicephalus* (*Boophilus*) *annulatus*. This Sahelian city and its region was Dori. 

The minimum sample size (*N*) was calculated following Flahault et al.’s [7] formula, which is:

(1) $$N=\frac{{\left[\left(Z_{\frac{\alpha }{2}}+\;Z_{\beta }\right)\sqrt{V\left(\theta \right)}\right]}^{2}}{L^{2}}$$

where $Z_{\frac{\alpha }{2}}$ is the upper $\frac{\alpha }{2}$ percentile of the standard normal distribution,$\;Z_{\beta }$ is the upper ß percentile of a standard normal distribution, 1-ß represents the wanted power, *L* the desired one-half width of CI (Confidence Interval), and $V\left(\theta \right)=Se\left(1-Se\right)$ is the variance function. *Se* is the sensitivity obtained via prior information or expert knowledge. 

Using the *Se* of 80% for the iELISA with a precision of +/–0.05, a usual power of 80%, α=0.05, $Z_{\frac{\alpha }{2}}=1.96$, $Z_{\beta }=0.84$; the result yields *N* = 502 cattle (total for northern, eastern, central, and southern parts of Côte d’Ivoire).

### 2.2. Laboratory Assays

For the molecular RT-PCR test, blood samples were collected on Whatman filter paper. Concerning the ELISA test, blood samples were taken in dried tubes. Then, serum was obtained by decantation from blood after 30 min to 1 h. Finally, the serum was stored at −20 °C until the serology assay. Molecular and serology tests were performed in parallel.

The RT-PCR assay was performed following the protocol as described by Gallego-Lopez et al. [8]. Samples positive to *Babesia bovis* and *Babesia bigemina* had, respectively, a Tm (melting temperature) of 76.43 °C and 74.15 °C. Concerning the iELISA test, it was performed following the protocol of Terkawi et al. [9]. The concentration of whole *Babesia bovis* antigen in each 100 µL well was 2 µg/mL. The whole antigen of *Babesia bovis* had been prepared and offered by an Australian partner (AusAID) via the Wecatic project that has been implemented by CIRDES (Centre International de Recherche sur l’Elevage en zone subhumid). CIRDES is situated in Burkina Faso. This antigen originated from cattle naturally infected by the Australian *Babesia bovis* strain. Briefly, when parasitaemia reached about 0.2%, blood samples were collected via the jugular vein on anticoagulant. Then, the blood was defibrinated, and red blood cells were suspended to a packed cell volume 5–10% of CO_2_ in 60% HEPES buffered medium with 40% bovine foetal serum. The pH was adjusted to 7 in the HCl solution. Then, it was transferred to a culture vessel for incubation in 5% of CO_2,_ at 37 °C, in 95% humidified air. Finally, every 48 h to 72 h, it was diluted from 3- to 25-fold by adding medium containing freshly collected uninfected cattle erythrocytes. The aim was to assess the test characteristics of iELISA with RT-PCR as the imperfect reference test. The sole modification concerned the positivity criteria. First of all, correcting optical density (OD) was completed for all samples encompassing positive and negative controls by subtracting white OD in their OD. Then, when a sample had a corrected OD superior to the positive control corrected OD, that sample was considered positive. Afterward, the threshold of positivity was compared to the positive control, which varied gradually as 5% (noted ELISA 5), 10% (noted ELISA 10), 15% (noted ELISA 15), 20% (noted ELISA 20), 25% (noted ELISA 25), 30% (noted ELISA 30), and 35% (noted ELISA 35). 

It is noteworthy that the iELISA and RT-PCR tests were performed by individuals blinded to the results of the other test.

### 2.3. Statistical Analysis

Different test characteristic parameters (sensitivity and specificity) were estimated. A Bayesian Latent Class Model (BLCM) was used to estimate the sensitivity (Se) and specificity (Sp) of iELISA [10]. The reason is that the Bayesian approach allows for the combination of both field data and prior expert information in the same model to estimate test characteristics. The second reason is that using a frequentist approach in an experimental design leads to a mild overestimate of parameters. Using prior information, a model was developed and run in Winbugs^®^ [11,12]. Additional calculations during the Bayesian analysis were performed in R (http://www.r-project.org) software. Three parameters were monitored during the analysis: the Deviance Information Criterion (DIC), the effective number of the estimated parameter (p_D_), and the Bayesian *p*-value (Appendix A, Table A1 and Table A2). Prior information for PCR was as follows: Se: (0.60–0.74); Sp: (0.90–1) [2]. Estimation was performed for different cut-off points, i.e., 5%, 10%, 15%, 20%, 25%, 30%, and 35%. The Receiver Operating Characteristic (ROC) curves and Area Under the Curve (AUC) were also determined using the RT-PCR as an imperfect reference test [13]. Correction of the ROC curve was conducted using the Neyman–Pearson criterion as described [14]. Moreover, the Youden Index (J) and Diagnostic Odds Ratio (DOR) Likelihood Ratios (positive and negative) were also estimated. The Youden Index was calculated as:

J = Se + Sp − 1 (2)

The Diagnostic Odds Ratio was estimated as:

DOR = (PPV/1 − PPV)/(1 − NPV)/NPV(3)

where PPV and NPV represent the Positive Predictive Value and the Negative Predictive Value. 

The Likelihood Ratios for a positive result (LR+) and for a negative result (LR−) were assessed following the respective equations:

LR+ = Se/(1 − Sp)(4)

LR− = (1 − Se)/Sp(5)

In addition, the comparison of AUC to 0.5 was conducted following these hypotheses:

Ho: AUC = 0.5, versus H1: AUC# 0, this test[(AUC − 0.5)/SE(AUC)] is normally distributed [15].

## 3. Results

Concerning the demography of the cattle population included in the study and effectively tested, in the north (Ferkessedougou, Korhogo, and Odienné), there were 323 cross-bred cattle of *Bos taurus* and *Bos indicus*, consisting of 197 dairy-cattle females and 126 males. There were 443 cattle of Girolando, cross-bred of *Bos taurus* and *Bos indicus* in the east (Agnibilekrou), west (Man), centre (Yamoussoukro), and south (Dabou, Abidjan). These 443 cattle consisted of 227 dairy-cattle females and 216 males. All of the cattle were adults from 2 years old to 11 years old. 

**Figure 2:**
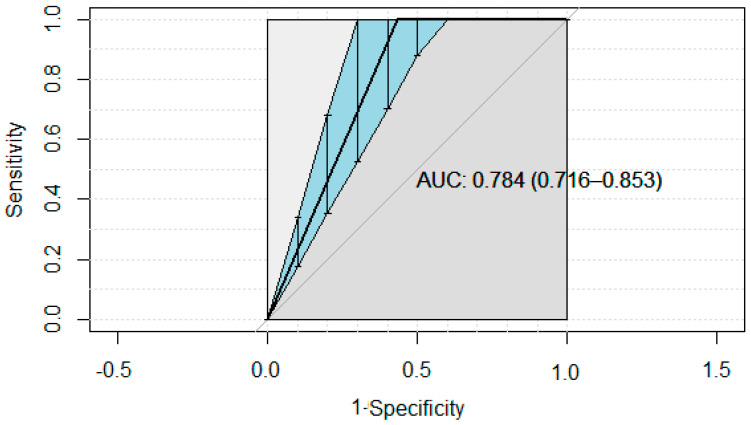
ROC curve of iELISA considering RT-PCR as the reference (AUC: Area Under the Curve).

The results of the sensitivity and specificity test are displayed (Table 1). The best cut-off suggested by the ROC curve (Figure 2) for the iELISA test was at 5% positivity. The ELISA test sensitivity and specificity were, respectively, 0.94 [95% Credibility interval (Cr. I.): 0.85–0.99] and 0.89 [95% Cr. I.: 0.87–0.91]. Positive and Negative Predictive Values for iELISA 5 were, respectively, 96.66% [92.71–100%] and 82.16% [72–93%] (Table 1). Then, with the following thresholds, Positive Predictive Values and Negative Predictive Values were, respectively, 94.58% [88–100%] and 63.73% [46–86.3%] (ELISA10); 93.52% [85.9–100%] and 52.43% [32.8–82.34%] (ELISA15); 94.37% [88.6–100%] and 49.65% [29.9–82.3%] (ELISA20). The area under the ROC curve (AUC) was 0.78 [0.72–0.85] for the iELISA test.

When considering the Youden Index (J) parameter, all of the values are greater than 0. So, for all of the thresholds (or at any cut-off), in general the iELISA correctly discriminated infected subjects from uninfected ones (Table 1) with a slight preference for the first cut-off ELISA5. Then, the Diagnostic Odds Ratio (DOR) variations (Table 1) showed the same trend with the optimal value of 127.85 [10.43–1562.27], demonstrating the best discriminatory power at ELISA5, followed by ELISA10 which had 31.7 [2–486.3] of DOR. For the rest of the thresholds, the DORs were around 15. According to the DOR parameter, it is noteworthy that the iELISA test is useful as long as their values are greater than 1. The Likelihood Ratio for a positive result, for all of the thresholds, indicated that infected subjects were very likely to be positive to the test compared to uninfected ones (Table 1). Similarly, the Likelihood Ratio for a negative result indicated that infected subjects were less likely to be positive to the tests compared to uninfected ones.

The monitoring of harmful effects following this diagnostic test study revealed one case of phlebitis that have been successfully managed. Before taking the sample, one of the cattle had an abscess on the left maxillary region that had been drained and treated. All of these veterinary examinations (including supplementary laboratory analyses) and cares were free of charge for the farmers.

## 4. Discussion

As in most studies of diagnostic test characteristics (mainly sensitivity and specificity), there is neither a gold standard test nor the availability of reliable information on subjects’ true status in the field (truly infected or truly uninfected). This study used a Bayesian framework to estimate iAb-ELISA test sensitivity and specificity. 

When choosing the optimal diagnostic threshold, six contexts could occur [1]. Amongst these situations, there are the demonstration of freedom from infection in an area, certifying freedom from infection for the trade or movement of animal(s), the estimation of infection prevalence or exposure in order to facilitate risk analysis or disease control, etc. The current study focused on the diagnostic test accuracy of iELISA. This situation compels the diagnostician to minimize misclassifications. Such misclassifications involve not only false negative subjects but also false positive ones. In consequence, the criterion of choice is maximum DOR. The reason is that DOR is independent of prevalence and the studied population. Therefore, its maximum value is a parameter of choice to select the cut-off [16] in molecular tests. In our study, ELISA 5 presented the maximum DOR and could be the best option. This is the first time, to the best of the authors’ knowledge, that the usefulness of DOR criterion in a serology test has been shown. Nevertheless, it is rare to get one perfect criterion diagnostic test for many reasons, for example, due to the complexity of accurately defining the limit between healthy subjects and diseased ones. Thus, analysing other diagnostic test characteristics such as maximum J index, maximum PPV and NPV, and the couple specificity-sensitivity could reveal critical facts. 

This is the first use of a Bayesian methodology for the performance of diagnostic tests for vector-borne diseases in Africa. Contrary to the frequentist methodology, this method eliminates the dependence on ROC curve to study population and prevalence hence [15]. The area under the curve of 0.78 [0.72–0.85] for iELISA demonstrates that it is a moderately accurate test according to the appropriate scale [17]. Additionally, the AUC shows that the test is significantly useful (*p* < 10^−4^). Indeed, in this scale, AUC = 0.5 means it is a non-informative test; 0.5 < AUC ≤ 0.7 refers to a less accurate test; 0.7 < AUC ≤ 0.9 corresponds to a moderately accurate test; 0.9 < AUC ≤ 1 describes a highly accurate test; and AUC = 1 refers to a perfect test. Then, as a principle, the optimal point on the ROC curve has to be closer to the left corner of the ROC curve. A strong advantage of this point is the high independence of the prevalence measurement from sensitivity and specificity [15]. 

Furthermore, the Youden Index maximizes the test accuracy when prevalence is 50%, which leads to a disadvantage of our test whatever the threshold choice [18], because of a prevalence of 76.4% [73.3; 79.3%]. This is clearly a limitation of the Youden Index criteria use in multiple threshold test comparisons. This high prevalence could be related to the farms surveyed which are located in favourable environments for ticks’ vectors such as *Rhipicephalus* (*Boophilus*) *microplus*. This tick plays a role in maintaining infection amongst cattle populations. Indeed, many visited localities are parts of humid climate areas in Côte d’Ivoire (more than 950 mm of annual rainfall). However, it is worth noting that several factors could influence cattle infection such as the infection rate of vector, the layout of tick and tick-borne disease control strategies, cattle acquired or innate immunity against ticks’ successful blood-feeding and parasites, cattle breed, number of neighbouring farms [19], and misclassifications (false negative and false positive, or in other words, misdiagnosis) due to the diagnostic test.

### 4.1. Probability of Misclassification Given an Infectious Status

Taking into account the maximum LR (+), the optimal threshold test was ELISA5. Then, when considering the minimum LR (−), ELISA5 was also the best threshold. The probability of misclassification given the infection status was 16.5%. This fact was highly comprehensive due to the best DOR at 127.85. Regarding the best second threshold in terms of discriminatory power (ELISA 10), the probability of misclassification given the infection status reached 30.6%. 

### 4.2. Probability of Misclassification Given a Test Result

If we have a positive test, the maximum PPV was at ELISA5. So, the probability of misclassification equalled 3.34% for positive results. It is noteworthy that all of the PPVs were superior to 93% whatever the threshold. In the case of a negative result, ELISA5 and ELISA10, with the maximum NPV of, respectively, 83% and 63%, have shown a high probability of misclassification for a negative result ranging from 17% to 37%. Here, since all of these NPVs were very close, it is not possible to distinguish the best threshold. This issue was recently addressed by maximizing the NPV, and then by privileging the better PPV [20]: these findings depicted the cut-off ELISA 5.

Nonetheless, in the best thresholds (ELISA5 and ELISA10), the probability of misclassification for a negative result is far from negligible (17% to 37%). These results clearly prove that this iAb-ELISA test seems more suited to situations of great prevalence because of less confidence in too many negative results, whereas this test affords sound confidence in positive results. Indeed, there is great confidence in subjects classified as positive to the test. In an experimental context, the competitive ELISA has been proven to be superior to iAb-ELISA [21]. This type of cELISA targeting on MSA (Merozoite Surface Antigen)-2c epitope of *Babesia bovis* has been shown to be useful in a prevalence study. Another limitation of this study is that cELISA performances (sensitivity and specificity, respectively, of 96.2% and 98%, but 89% and 96% when tested with field samples) have been compared to IFAT. Then, the ROC curve was the unique tool (despite its drawback of being prevalent dependent if the cut-off is not optimal) chosen in their survey to select its best cut-off that equalled to a PI (percentage of inhibition) of 29.5. 

Likewise, in South Africa, another seroprevalence survey of cattle babesiosis due to *Babesia bovis* and *Babesia bigemina* using iELISA and IFAT reported on the suitability of iELISA targeting on recombinant spherical body protein-4(BbSBP-4) for *B. bovis* and C terminal rhoptry-associated protein-1(BbigRAP-1/CT) for *B. bigemina*. Indeed, this study’s conclusion is based on the good agreement between iELISA and IFAT for the detection of *B. bovis* (91.26%) and *B. bigemina* (84.01%) [22]. One major concern regarding this conclusion is that a good agreement can exist between an index test and a reference test, but it could be possible that the index test is not good. 

Amongst the limitations of the current study, the RT-PCR failed to detect carrier animals, probably due to low parasitaemia, while the iELISA test performed better. In a contrary situation, there were five cattle classified as negative to iELISA but positive to the RT-PCR and MGG test. In reality, these subjects must be seropositive. We can explain these defaults of negativity to well-performed iELISA by immunosuppression (substantiated by leukopenia associated or not with anaemia), and clinically shown their long-lasting (more than 6 months) poor Body Condition Scoring (BCS) that was between 2 and 4 on a scale of 1 to 9. In a follow-up, there were three confirmed cases of TB, one confirmed case of paratuberculosis, and one confirmed case of chronic Oesophagostomosis (an intestinal chronic parasitic disease). There were situations in which 11 dairy-cattle that were in the stable 2 months prior carried no ticks (so zero grazing), and were RT-PCR negative but positive to iELISA. After investigation, they had been treated with diminazene aceturate (pharmacologically active against *Trypanosoma congolense*, *Trypanosoma vivax*, *Babesia bovis*, and *Babesia bigemina*) more than 7–10 weeks before. According to the index iELISA test being evaluated, these subjects were classified as infected, but they were not in reality. During the study timeline, they were healthy cattle that were still seropositive because of a past successful cure of *Babesia bovis* infection. Moreover, this study raised another dilemma: considering that the indirect immunofluorescence antibody test (IFAT) has been the reference test until recently, what are its comparative performances in similar field conditions? For further research, we would recommend the development of an ELISA that can detect antibodies against non-structural proteins of the parasite that are released in the blood during infection. 

To the best of the authors’ knowledge, the current work is the first study on the assessment of diagnostic test performance using BLCM, and its updated indications concerning animal vector borne disease. Besides animal veterinary records or condition, disease stage, and entomological factors, the complexity of finding the true infection status of the subject increases with these vectorial diseases: babesiosis could be in an epidemic situation, or endemic stability, or endemic non-stability. 

Unfortunately, many authors and national veterinary authorities have used this test without knowing its accuracy and subsequently announced high prevalence.

## 5. Conclusions

The current study highlighted the performance of the iAb-ELISA test in the realm of the field. Amongst the seven thresholds, ELISA5 or ELISA10 appeared to be the best options. The DOR could be amongst the criteria of choice in serology test diagnostic accuracy. For instance, this test could be appropriate in assessing moderate prevalence. The ranges of moderate and high prevalence have to be addressed. This issue is specifically relevant in the case of the eradication strategy framework that includes many steps. Such steps are defined following the prevalence level. Further study is needed to demonstrate whether the sibling c-ELISA test is best when we do not know the true status of animals in the field. 

## Figures and Tables

**Table 1:** iELISA test characteristics according to threshold positivity.

Test Threshold Positivity	Se	95% Cr. I.	Sp	95% Cr. I.	NPV (%)	PPV (%)	J	DOR	LR+	LR−
ELISA5	0.940	0.854–0.998	0.895	0.871–0.919	82.16	96.66	0.835	127.85	8.95	0.07
ELISA10	0.852	0.767–0.916	0.842	0.263–0.993	63.73	94.58	0.694	31.7	5.39	0.17
ELISA15	0.768	0.689–0.838	0.828	0.201–0.994	52.43	93.52	0.596	15.93	4.46	0.28
ELISA20	0.731	0.650–0.796	0.859	0.252–0.996	49.65	94.37	0.59	16.71	5.18	0.31
ELISA25	0.675	0.599–0.741	0.864	0.253–0.996	41.57	93.7	0.539	13.4	4.96	0.37
ELISA30	0.623	0.545–0.688	0.910	0.383–0.998	42.71	95.72	0.533	16.87	6.92	0.41
ELISA35	0.566	0.492–0.626	0.918	0.438–0.998	39.51	95.71	0.484	14.68	6.90	0.47

Footnotes: Se: Sensitivity; Sp: Specificity; NPV: Negative Predictive Value; PPV: Positive Predictive Value, DOR: Diagnostic Odds Ratio; LR+: Likelihood Ratio for positive test; LR−: Likelihood Ratio for negative test; J: Youden Index.

## Appendix A

**Table A1:** Bayesian mathematical study.

	Node	Mean	Sd	MC Error	2.5%	Median	97.5%	Start	Sample
	bayesp	0.5297	0.4991	0.001378	0.0	1.0	1.0	5001	150,000
	p[1]	0.03204	0.006148	1.566 × 10^−5^	0.0211	0.03166	0.04509	5001	150,000
	p[2]	0.003103	0.001889	6.4 × 10^−6^	5.681 × 10^−4^	0.002731	0.007777	5001	150,000
	p[3]	0.1131	0.01114	2.883 × 10^−5^	0.09222	0.1127	0.1358	5001	150,000
	p[4]	0.01634	0.004399	1.067 × 10^−5^	0.008867	0.01595	0.026	5001	150,000
	p[5]	0.07919	0.009465	2.57 × 10^−5^	0.06164	0.07886	0.09857	5001	150,000
	p[6]	0.02073	0.004975	1.29 × 10^−5^	0.01214	0.02033	0.03156	5001	150,000
	p[7]	0.5261	0.01756	4.726 × 10^−5^	0.4916	0.5261	0.5604	5001	150,000
	p[8]	0.2095	0.01435	3.649 × 10^−5^	0.182	0.2092	0.2383	5001	150,000
PCR	se[1]	0.917	0.05656	0.001267	0.8282	0.9192	0.998	5001	150,000
FS	se[2]	0.8743	0.01694	1.349 × 10^−4^	0.8402	0.8746	0.9058	5001	150,000
Elisa5	se[3]	0.2616	0.0203	2.625 × 10^−4^	0.2238	0.261	0.3025	5001	150,000
PCR	sp[1]	0.9484	0.02894	9.576 × 10^−5^	0.9023	0.9477	0.9972	5001	150,000
FS	sp[2]	0.2976	0.2267	0.003191	0.02554	0.2299	0.883	5001	150,000
Elisa5	sp[3]	0.7946	0.172	0.002593	0.275	0.8569	0.9616	5001	150,000

**Table A2:** Dbar = post.mean of −2logL; Dhat = −2LogL at post.mean of stochastic nodes.

	Dbar	Dhat	pD	DIC
Res	42.332	57.595	−15.264	27.068
th	4.605	4.605	0.000	4.605
total	46.937	62.200	−15.264	31.673
